# Indeterminate (B3) Breast Lesions and the Ongoing Role of Diagnostic Open Biopsy

**DOI:** 10.1155/2021/5555458

**Published:** 2021-12-27

**Authors:** Elizabeth Tan, Asiri Arachchi, Michael Cheng, Darren Lockie

**Affiliations:** ^1^Monash Health, Australia; ^2^Eastern Health, Australia

## Abstract

**Introduction:**

Due to their uncertain malignant potential, indeterminate breast lesions on core needle biopsy (CNB) require diagnostic open biopsy (DOB). This study evaluated DOB results given largely benign pathology. Lesions included are atypical papilloma, atypical ductal hyperplasia (ADH), atypical lobular hyperplasia (ALH), and radial scar/complex sclerosing lesions (RS/CSL). *Methodology*. A retrospective audit from 2010 to 2017 analysed patients with a screen-detected suspicious lesion and indeterminate (B3) CNB diagnosis. Primary outcome was the malignancy upgrade rate, with secondary evaluation of patient factors predictive of malignancy including age, symptoms, mammogram characteristics, lesion size, biopsy method, and past and family history.

**Results:**

152 patients (median age 57 years) were included, with atypical papillomas being the largest subgroup (44.7%). On DOB histology, 99.34% were benign, resulting in a 0.66% malignancy upgrade rate. Patient characteristic analysis identified 86.84% of B3 lesions were in patients greater than 50 years old. 90.13% were asymptomatic, whilst 98.68% and 72.37% had a negative past and family history. Majority 46.71% of lesions had the mammogram characteristic of being a mass. However, with 57.89% of the lesion imaging size less than 4 mm, a corresponding 60.5% of core needle biopsies were performed stereotactically. The small malignant subgroup limited predictive factor evaluation.

**Conclusion:**

Albeit a low 0.66% malignancy upgrade rate in B3 lesions, no statistically significant patient predictive factors were identified. Until predictive factors and further assessment of vacuum-assisted excision (VAE) techniques evolve, DOB remains the standard of care.

## 1. Introduction

Increased CNBs on screen-detected suspicious lesions have led to more indeterminate (B3) lesions. These proliferative lesions are histologically classified as B3 due to their uncertain malignant potential and heterogeneity [1–3]. The positive predictive value for malignancy is 25-30%, though each lesion's malignancy risk varies significantly [1–4].

Historically, it was standard practice to perform a diagnostic open biopsy (DOB), due to the risk of malignancy underestimation [1–5]. However, this is debated due to improved radiological imaging modalities, biopsy techniques, and majority of B3 lesions benign excisional histology [1, 3–5]. This has resulted in increased overtreatment and health costs [1, 3]. This retrospective study is aimed at assessing metropolitan eastern Victoria's malignancy upgrade rate for B3 lesions on final histology and positive predictive value of patient factors.

## 2. Methods

A single-institution retrospective review was conducted over an eight-year period (April 2010–September 2017). The 3 inclusion criteria were patients diagnosed with Breast Imaging Reporting And Data System (BIRADS) category 4 lesions, subsequent indeterminate histology (B3) on CNB, and DOB. The 4 specific B3 histology lesions identified from the Breast Screen database were atypical papillomas, atypical ductal hyperplasia (ADH), atypical lobular hyperplasia (ALH), and radial scar/complex sclerosing lesion (RS/CSL). To ensure consistency, all histology was reviewed by 2 pathologists at the same pathology lab and discussed at our multidisciplinary team meeting.

The primary outcome assessed was malignancy upgrade rate, with secondary outcomes on potential factors associated with the likelihood of carcinoma. These included age, presence or absence of symptoms, imaging characteristics of the lesion, biopsy method, and size.

## 3. Results

152 patients were identified, with 132 (86.84%) of the study population age greater than 50 years old. Majority 137 patients (90.13%) were asymptomatic. The incidence and final histology of the 4 lesion types—atypical papilloma, ADH, ALH, and RS/CSL—are demonstrated in Table 1. CNBs were performed on mass lesions using ultrasound-guided 14-gauge core needle, whilst microcalcification core biopsies were performed with 9-gauge vacuum-assisted biopsy (VAB) under stereotactic or tomosynthesis guidance.

The most common radiographic abnormality was a mass, present in 46.71% of cases. 57.89% of all B3 lesions were less than 6 mm. On final excisional histology, 151 lesions were benign, with only 1 ductal carcinoma in situ (DCIS) lesion. They had the associated probability values of 94.12 and 5.88, respectively. Zero patients were lost to follow-up.

Table 2 demonstrates the limited analysis conducted on 7 potential patient factors predictive of malignancy. Further statistical analysis of the positive predictive values using Fisher's test and *χ*^2^ test according to Pearson was negated by the small malignancy upgrade rate.

## 4. Discussion

According to the Cancer Council and Australia Institute of Health and Welfare [6, 7], the incidence of new breast cancer diagnoses increased from 5,374 in 1982 to 17,004 in 2015, becoming the most commonly diagnosed cancer in females and second most common cause of death from cancer among females. Despite this, the improved five-year survival rate between 1985-1989 and 2011-2015 from 74% to 91% is attributed to advanced imaging techniques and the introduction of Breast Screen—leading to early cancer detection and treatment [6, 7]. However, with an increased number of radiologically suspicious lesions, subsequent CNB decreased the number of DOB for previously unknown B1 and B2 lesions. A diagnostic dilemma remains for B3 lesions, defined as a heterogeneous lesion with associated atypia [1–5]. The B3 lesions encompassed in this study are papillomas, ADH, ALH, and RS/CSL.

DOB is currently recommended in B3 lesions due to their uncertain malignant potential and association with invasive carcinoma or DCIS [1–5]. The second reason is the heterogeneity of the lesions, with CNB sample not characteristic of the whole lesion, with potential risk of coexisting in situ or invasive carcinoma [1, 3, 4]. Hence, traditional further evaluation with a DOB was recommended to accurately establish the diagnosis and management plan [2].

With the malignancy positive predictive value for B3 lesions at 25-30%, this varies according to lesion subcategory [1]. However, the larger bore CNB and increased target lesion sampling have decreased the malignancy upgrade rate [1–3]. Issues arose of overtreatment in benign disease, accuracy of nonoperative diagnoses, and health costs of increased DOB [1, 2]. The literature also highlights DOB-associated risks such as vasovagal reactions secondary to hookwire-localized biopsy procedures, anaesthetic complications, infections, haematomas, and abscess formation [4]. These concerns have caused ongoing controversy, with some authors suggesting surveillance in certain subset groups [1–4, 8–12].

### 4.1. Primary Outcome: Malignancy Upgrade Rate

Out of 152 patients, 151 lesions were benign (99.34%), with 1 DCIS. This 0.66% malignancy upgrade rate demonstrates that an overwhelming majority of our diagnostic open biopsies were benign. Like Conlon et al.'s study [13], this low malignancy upgrade rate is likely secondary to thorough radiological and pathological correlation, as well as procedurally advanced techniques of CNB. We postulate the smaller CNBs in the previous century obtained smaller and fewer sample sizes, resulting in indeterminate (B3) lesions [1–3]. The diagnostic accuracy was increased with the advancement of larger core biopsy needle sizes and targeted biopsies of the indeterminate lesion's peripheral border and centre [1–4]. Hence, we feel that this increased accuracy of core needle biopsies should lead to a corresponding reduction in the incidence of DOB.

### 4.2. Atypical Papilloma

The largest subcategory of B3 lesions in our study's CNB group was atypical papilloma, with 44.7% of our patients. Characterised by a fibrovascular core with overlying epithelium into ductal lumens [5], they have an associated 67% risk of malignancy [1]. Disproportionally, our subgroup had a malignancy upgrade rate of 0%. Bianchi et al. [5] further classified the upgrade rate in a papilloma without atypia to be 9-13.2%, whilst higher with a rate of 36-47.8% in the presence of atypia.

### 4.3. ADH

Atypical hyperplasia is subcategorised into ADH and ALH, both lesions sharing some cytological features of low-grade DCIS and LCIS, respectively [14–16]. They confer an increased risk of breast cancer (RR 3.7 to 5.3) [16]. Whilst defined as a localized intraductal proliferation [17–19], foci of ADH can also be present at the periphery of DCIS lesions [16, 19]. Molecular studies postulate that ADH may represent clonal neoplastic proliferations, which would suggest it as a precursor to cancer [19]. With limited CNB tissue sample, it is difficult to distinguish ADH from DCIS on histopathology. The upgrade rate of ADH to in situ and invasive carcinoma on DOB varies widely from 7 to 87% [16, 18]. This is likely due to the differences in pathological diagnosis thresholds on CNB [16]. Our study had 47 ADH lesions with a surprising 0% malignancy upgrade rate. Pena et al.'s study [18] classified ADH lesions into a low- and high-risk subset, based on number of ADH foci, percentage of imaging lesion removed, and lack of cell necrosis. It postulated that women in these low-risk criteria could be considered for surveillance; however, the risk required to avoid DOB is still unclear.

### 4.4. RS/CSL

RS is defined by a stellate pattern of a fibroelastic core with radiating ducts, whilst complex sclerosing lesions are radial scars greater than 1 cm in dimension [13, 20, 21]. Like ADH and ALH, the challenging architectural distortion can mimic low-grade carcinoma [20]. Jacobs et al.'s case control study [20] reported that radial scars had twice the risk of breast cancer, with the risk even larger with larger radial scars (CSL) or multiple lesions. Conlon et al. [13] reported an upgrade malignancy rate of 2% on surgical excision, whilst Rakha et al. [2] reported a 9% upgrade rate in 410 radial scars without atypia on CNB, whilst those with atypia had a rate of 36%. Once again, our 0% malignancy upgrade rate in RS/CSL is lower than the literature.

### 4.5. ALH

The final and smallest subcategory is ALH, defined as an intralobular epithelial proliferation of discohesive cells with decreased or absent E-cadherin expression on immunohistochemistry [1, 14]. Although Hussain's systematic review [14] has demonstrated an upgrade rate of 27%; there is new evidence that the upgrade risk is low (less than 5%) if there is imaging-pathologic concordance [12]. With the small incidence of 2.63% in our study group, we cannot comment on our 25% malignancy upgrade rate. However, the American Society of Breast Surgeons [12] no longer recommends routine excision of ALH if two factors are present, radiological-pathological concordance and the absence of other atypical lesions such as ADH, papilloma, or radial scar.

### 4.6. Secondary Outcome of Patient Factors Predictive of Malignancy

The only malignant lesion on DOB was identified in the group of patients more than 51 years old. Most studies [15–17, 21] observed our findings, with an increase in underestimation rates in patients aged 50 years and above. Both Ko et al. [17] and Chae et al.'s [16] ADH studies also noted lesion size > 15 mm and >10 mm, respectively, to be statistically significant as well. Forgeard et al.'s retrospective analysis of 300 patients found that there was a low estimation rate of 4% in groups with lesion size < 6 mm and ≤2 foci ADH in microcalcifications. However, they found a malignancy rate of 36 to 38% in lesions ≥ 21 mm, <6 mm lesions with incomplete removal, or >2 foci ADH in microcalcifications [8]. The number of foci of ADH as a predictor of malignancy is also echoed in Pena et al.'s study of 399 cases [18]. This contradicts our findings as our malignant lesion was in the subgroup with an imaging size less than or equal to 5 mm. A majority 46.71% of total lesions were characterised as a mass on mammogram; however, our malignant lesion was a microcalcification. Rakha et al.'s study had similar results to us, with a higher incidence of malignancy in their screen-detected calcifications (40%) compared to mass lesions and architectural distortion [2].

Although Forgeard et al. [8] recommended surveillance for his first 2 subset patient groups (<6 mm lesions and ≤2 foci ADH in microcalcifications), we found that the factors of imaging size and mammogram characteristics could not safely predict the absence of DCIS or invasive carcinoma.

With the CNB modality subgroup, we noticed a rising trend in more stereotactic (92/152 = 60.5%) than ultrasound (60/152 = 39.5%)-guided core needle biopsies, with (21%) more in the former category. The stereotactic core biopsies were also performed with a larger 9-gauge needle, in comparison to the 14 G needle on ultrasound-guided biopsies. As our data was from a screening program of majority asymptomatic patients, it was unsurprising that the malignant lesion was from the asymptomatic, negative past and family history groups. Hence, this management pathway has been debated with some authors [2, 4, 13, 15, 17], viewing DOB as an unnecessary intervention for a mostly benign lesion, particularly in asymptomatic women.

Despite our results in Table 2, none of the 7 patient factors we identified—age, presence or absence of symptoms, imaging characteristics of the lesion, biopsy method, and size—were statistically predictive of malignancy. We found no correlation in predicting the likelihood of carcinoma if a patient had these factors versus a patient without. This finding, similar to Conlon et al.'s study [13], is likely attributed to our very low malignancy upgrade rate; hence, the analysis of these factors was limited.

Rakha et al. [2] found that lesion removal by vacuum-assisted excision (VAE) in selected cases may be a safe option. The United Kingdom National Health Service (UK NHS) guidelines have also recommended that B3 lesions on CNB should undergo further management with VAE. This increases the likelihood of sampling adjacent heterogeneity or possible malignant cells, as well as complete target excision [1]. This diagnostic technique is relatively safe and cost-effective and reduces the number of benign surgical excisions [1, 4, 12]. The only recommended B3 lesions for ongoing DOB were papillary lesions with atypia and lesions difficult to diagnose histologically [1]. Three UK units have followed this new management pathway [9–11], with Strachan et al. performing a secondary biopsy with VAE in 321 of 398 B3 lesions [9]. 24% of those patients required surgical excision, whilst 245 avoided surgery, with zero patients having cancer at the biopsy site at their 3-year follow-up [9].

VAE has also been used in Europe and the United States of America (USA) in the management of B3 lesions with no atypia [4, 12]. The first international consensus conference in 2016 recommended a therapeutic VAE excision for CNB-confirmed lobular neoplasia lesions, papillomas, and RS [3]. However, American and international guidelines recommended DOB for ADH due to inconclusive data [3, 12]. They also recommended DOB in cases of CNB pathology-imaging discordance [12] and other lesions with atypia such as atypical papillomas [4].

With vacuum-assisted techniques already being performed as a CNB in the Australian setting, it could progress to a feasible option as a minimally invasive management pathway. As an outpatient procedure, VAE with a 7-8 G needle would provide equivalent number of samples to a DOB and complete excision of lesions less than 15 mm [4]. Hence, surgical complications and postsurgical scarring impacting surveillance imaging can be avoided [4]. Although we had a low postoperative complication rate of 1.97%, 1.32% of our patients underwent a second diagnostic open biopsy due to clip migration.

Another reason albeit economical is the financial savings of therapeutic VAE compared to a DOB [1, 3]. We postulate that the VAE device implementation and day case procedure would be significantly less than the procedural DOB costs of preoperative imaging, theatre time, overnight admission, and postoperative follow-up. This combined VAE and surveillance plan would minimise the number of benign DOB and at the same time therapeutically excise malignancies. Annual mammogram surveillance is subsequently recommended for 5 years [3, 4, 9, 12], with more intense surveillance for lobular neoplasia [3]. This postprocedural plan would be similar to Breast Screen's current policy, which also emphasises education on breast care awareness and regular clinical examinations.

The authors acknowledge the limited sample size of 152 patients and potential selection bias of only analysing certain B3 lesions and patient factors. Patient factors predictive of malignancy could not be assessed and were deemed to be statistically insignificant due to only 1 malignant lesion identified. This could have been rectified with a larger population group including all B3 lesions or a longer time period. Data on the differences in ultrasound-guided and stereotactic core needle biopsies such as the number of cores per biopsy could have been analysed to see if that was a predictor of malignancy.

## 5. Conclusions

Despite advances with imaging and CNB, our understanding on the malignant potential of high-risk B3 lesions such as atypical papilloma, ADH, ALH, and RS/CSL lesions is still limited. Our preliminary study found a low 0.66% malignancy upgrade rate; hence, we conclude that not all B3 lesions should undergo diagnostic open biopsy. However, in view of our lack of statistically significant patient factors to accurately predict the rate of upstaging, DOB is still an essential component in the assessment of B3 breast lesions. Further studies are needed to identify clinical, radiological, and pathological parameters in a recommendation for DOB versus VAE—a management pathway increasingly used in other countries. It may likewise help reduce our benign DOB rate. The management of indeterminate breast lesions is an ongoing dilemma, with a case-by-case multidisciplinary team approach and more research needed to guide clinical management.

## Figures and Tables

**Table 1:** Malignancy upgrade rate for each B3 lesion subtype.

B3 lesion on CNB	Incidence (%)	Benign on surgical excision	Malignant on surgical excision	Malignancy upgrade rate (%)
Atypical papilloma	68 (44.70%)	68	0	0
ADH	47 (30.90%)	47	0	0
ALH	4 (2.60%)	3	1	25
Radial/(CSL)	33 (21.70%)	33	0	0
Total	152	151	1	0.66

**Table 2:** Patient characteristics/factors predictive of malignancy.

Factors	*N* = 152	Benign	Malignant
*Age*			
≤50	21	21	0
≥51	131	130	1
*Symptomatic*			
Positive	16	16	0
Negative	136	135	1
*Family history*			
Positive	42	42	0
Negative	110	109	1
*Imaging size*			
≤5 mm	88	87	1
5-10 mm	30	30	0
≥11	34	34	0
*CNB modality*			
Ultrasound 14 G	60	60	0
Stereotactic 9 G	92	91	1
*Mammogram characteristics*			
Mass	71	71	0
Distortion	14	14	0
Microcalcification	67	66	1
*Past history of breast cancer*			
Positive	2	2	0
Negative	150	149	1

## Data Availability

The nature of the data is patient and tumour characteristics at our institution; hence, access is restricted due to legal and ethical concerns of patient privacy. However, the data is available from the corresponding author upon request.

## References

[1] Pinder S. E., Shaaban A., Deb R. (2018). NHS Breast Screening multidisciplinary working group guidelines for the diagnosis and management of breast lesions of uncertain malignant potential on core biopsy (B3 lesions). *Clinical Radiology*.

[2] Rakha E. A., Lee A. H. S., Jenkins J. A., Murphy A. E., Hamilton L., O’Ellis I. (2011). Characterization and outcome of breast needle core biopsy diagnoses of lesions of uncertain malignant potential (B3) in abnormalities detected by mammographic screening. *International Journal Cancer*.

[3] Rageth C. J., O’Flynn E. A. M., Comstock C. (2016). First International Consensus Conference on lesions of uncertain malignant potential in the breast (B3 lesions). *Breast Cancer Research and Treatment*.

[4] Shaaban A. M., Sharma N. (2019). Management of B3 lesions—practical issues. *Current Breast Cancer Reports*.

[5] Bianchi S., Bendinelli B., Saladino V. (2015). Non-malignant breast papillary lesions- B3 diagnosed on ultrasound-guided 14-gauge needle core biopsy: analysis of 114 cases from a single institution and review of the literature. *Pathology Oncology Research*.

[6] Australian Institute of Health and Welfare (2020). *Cancer in Australia 2019. Cancer series no. 119. Cat. no. CAN 123*.

[7] Thursfield V., Farrugia H. (2017). *Cancer in Victoria: Statistics and Trends 2016*.

[8] Forgeard C., Benchaib M., Guerin N. (2008). Is surgical biopsy mandatory in case of atypical ductal hyperplasia on 11-gauge core needle biopsy? A retrospective study of 300 patients. *American Journal of Surgery*.

[9] Strachan C., Horgan K., Millican-Slater R. A., Shaaban A. M., Sharma N. (2016). Outcome of a new patient pathway for managing B3 breast lesions by vacuum-assisted biopsy: time to change current UK practice?. *Journal of Clinical Pathology*.

[10] Rajan S., Shaaban A. M., Dall B. J., Sharma N. (2012). New patient pathway using vacuum-assisted biopsy reduces diagnostic surgery for B3 lesions. *Clinical Radiology*.

[11] Pieri A., Hemming D., Westgarth J., Lunt L. (2017). Vacuum-assisted biopsy is a viable alternative to surgical biopsy in the investigation of breast lesions of uncertain malignant potential. *The Surgeon*.

[12] Schnitt S. J. (2019). Problematic issues in breast core needle biopsies. *Modern Pathology*.

[13] Conlon N., D’Arcy C., Kaplan J. B. (2015). Radial scar at image-guided needle biopsy: is excision necessary?. *The American Journal of Surgical Pathology*.

[14] Hussain M., Cunnick G. H. (2011). Management of lobular carcinoma in-situ and atypical lobular hyperplasia of the breast-A review. *European Journal of Surgical Oncology*.

[15] McGhan L. J., Pockaj B., Wasif N., Giurescu M. E., McCullough A. E., Gray R. J. (2012). Atypical ductal hyperplasia on core biopsy: an automatic trigger for excisional biopsy?. *Annals of Surgical Oncology*.

[16] Chae B. J., Lee A., Song B. J., Jung S. S. (2009). Predictive factors for breast cancer in patients diagnosed atypical ductal hyperplasia at core needle biopsy. *World Journal of Surgical Oncology*.

[17] Ko E., Han W., Lee J. W. (2008). Scoring system for predicting malignancy in patients diagnosed with atypical ductal hyperplasia at ultrasound-guided core needle biopsy. *Breast Cancer Research and Treatment*.

[18] Pena A., Shah S., Fazzio R. (2017). Multivariate model to identify women at low risk of cancer upgrade after a core needle biopsy diagnosis of atypical ductal hyperplasia. *Breast Cancer Research and Treatment*.

[19] Lakhani S. R., Collins N., Stratton M. R., Sloane J. P. (1995). Atypical ductal hyperplasia of the breast: clonal proliferation with loss of heterozygosity on chromosomes 16q and 17p. *Journal of Clinical Pathology*.

[20] Jacobs T. W., Byrne C., Colditz G., Connolly J. L., Schnitt S. J. (1999). Radial scars in benign breast biopsy specimens and the risk of breast cancer. *The New England Journal of Medicine*.

[21] Doyle E. M., Banville N., Quinn C. M. (2007). Radial scars/complex sclerosing lesions and malignancy in a screening programme: incidence and histological features revisited. *Histopathology*.

